# Coping Strategies and Posttraumatic Growth Following Transient Ischemic Attack: A Qualitative Study

**DOI:** 10.3390/jcm12020575

**Published:** 2023-01-10

**Authors:** David Kindermann, Veronika Maria Grosse-Holz, Martin Andermann, Peter Arthur Ringleb, Hans-Christoph Friederich, Timolaos Rizos, Christoph Nikendei

**Affiliations:** 1 Department of General Internal Medicine and Psychosomatics, University Hospital Heidelberg, University of Heidelberg, 69120 Heidelberg, Germany; 2 Department of Neurology, University Hospital Heidelberg, University of Heidelberg, 69120 Heidelberg, Germany

**Keywords:** coping strategies, posttraumatic growth, transient ischemic attack

## Abstract

A transient ischemic attack (TIA) is defined as a temporary neurological dysfunction due to focal brain ischemia. We aimed to identify common coping strategies and the possible occurrence of posttraumatic growth in TIA patients. Semistructured interviews were conducted with TIA patients three months after TIA. We asked the participants about possible changes in the aftermath of their TIA and their way of coping with said changes. All interviews were tape-recorded and subsequently transcribed verbatim. Thematic content analysis was performed to identify main categories and themes. Seventeen patients with a median age of 66 years completed the semistructured interviews. Qualitative content analysis revealed 332 single codes, from which the three main categories “impairments as a consequence of TIA”, “coping strategies” and “posttraumatic growth” were generated. The main categories were further subdivided into seven categories and thirty-six themes. TIA patients may suffer from various physical impairments, which also involve medication side effects. Activating resources on the one hand, and avoiding negative thoughts and feelings on the other hand, were identified to be the relevant coping strategies in TIA patients. Posttraumatic growth seems to be a common phenomenon after TIA, which may have important implications for treatment and rehabilitation.

## 1. Introduction

Stroke is the second leading cause of death in Germany and worldwide and a major cause of disability in adulthood [1]. Even though incidence and mortality rates in Germany have steadily decreased over the past decades, the absolute number of people affected by stroke is continuously increasing due to demographic trends [1,2]. One frequent precursor of a stroke is a transient ischemic attack (TIA). According to the 2009 statement of the American Heart Association (AHA) and the American Stroke Association (ASA), a TIA can be defined as a transient neurological dysfunction due to focal brain ischemia, that persists for 24 h or less [3]. The underlying temporary ischemia in TIA is not associated with acute infarction and may manifest as sensory disturbances, paralysis, or speech, and visual disturbances, for example. With an estimated lifetime risk of 5:1000, TIAs represent a highly frequent neurological emergency condition [4]. The occurrence of a TIA could previously be identified as an important predictor for a subsequent stroke; in this context, it is assumed that a stroke occurs in 10–15% of cases after a TIA [5,6]. Due to its unpredictable and uncontrollable onset and its threat to physical integrity, the event of a TIA can be described as a traumatic event according to the DSM-5 criteria, which may subsequently lead to posttraumatic stress disorder (PTSD) [7].

Various studies have recently investigated the incidence of PTSD symptoms as a possible sequela of different medical conditions or treatments [8,9,10]. PTSD is characterized by the main symptoms of (a) intrusions or reexperience of sensory impressions of the traumatic event, (b) avoidance of stimuli or situations, associated with the traumatic event and (c) persistent vegetative hyperarousal, such as tachycardia, sleeping disorders, or irritability [11]. The prevalence of PTSD following stroke and TIA is estimated to be approximately 23% [9]. Studies differentiating the prevalence of PTSD after experiencing a stroke on the one hand and after TIA on the other hand are still rare. In a cross-sectional study, Kiphuth et al. found a prevalence rate of 29.6% for PTSD in TIA patients three months after a TIA incident [12]. In a recently published study of a total of 61 TIA patients, we found PTSD in 24.6% of all TIA cases after three months [7]. In this context, sociodemographic factors, psychiatric history, and social support were identified as important predictors for the development of PTSD after TIA [7]. Post-TIA PTSD has been shown to be associated with poor mental health outcomes and is suspected to decrease adherence to strategies of secondary prevention [13].

However, many people also indicate positive changes as a result of traumatic events. The concept of Posttraumatic growth (PTG) is defined as subjectively experienced positive changes after being confronted with traumatic events [14]. Examples of PTG include an increased appreciation for one’s life, increased experience of closeness in relationships, positive changes in priorities, recognition of personal strengths, or positive spiritual development [14]. With regard to neurological disorders, the phenomenon of PTG has been investigated mainly after traumatic brain injury and in multiple sclerosis [15]. In a study from 2007, Powell et al. examined the time course of PTG in patients after traumatic brain injury by comparing the level of PTG 1–3 years post-injury and 10–12 years post-injury [16]. A higher level of PTG was found in the latter group, which indicates that PTG after traumatic brain injury is not only a common phenomenon but also seems to increase over time [16]. A recent study by Gil-Gonzales et al. analyzed the longitudinal course of PTG in a group of patients with multiple sclerosis [17]. It was shown that the level of PTG increased over a follow-up period of 36 months; furthermore, the positive psychological changes were still present 12 years after the initial diagnosis [17]. In this context, a higher pain severity, female gender, and higher levels of anxiety could be demonstrated as positive predictors of PTG in multiple sclerosis [17]. However, the phenomenon of PTG does not appear to be specific to neurological disorders; in a 2016 study, the occurrence of PTG in patients after traumatic brain injury was compared to patients after myocardial infarction and similar levels were found in both disease entities [18]. The occurrence of PTG in stroke patients has been demonstrated in different studies [19,20,21]. In this context, stroke patients report, among other aspects, an increased appreciation of life and an intensification of relationships as a result of the stroke [21]. Previously, interview studies with TIA patients have partly revealed positive changes after TIA in addition to problems and limitations. For instance, TIA patients reported gratitude for the positive “warning signal” that the TIA represents for their health behaviors, as well as lifestyle changes implemented due to the TIA [22,23]. The previous qualitative studies on this topic chose a relatively open thematic framework. However, detailed qualitative investigations of the possible positive psychological changes in TIA patients are not yet available. Therefore, the present study aimed to conduct a detailed qualitative analysis of the positive changes in the sense of PTG after TIA.

In addition to analyzing positive changes in the sense of PTG after TIA, another aim of the present study was to identify specific coping strategies in TIA patients. The concept of coping was initially developed by Lazarus and describes a process in which an individual first examines the degree of threat of a stressor and the available resources in order to subsequently develop a specific strategy for dealing with the stressor [24]. In general, a distinction can be made between active and passive coping strategies [25,26]. Active strategies are, for example, obtaining information about a stressor, activating helpful resources, or attempting to control the stressor, whereas passive strategies describe, for example, withdrawing from and enduring the stressor [26,27]. Furthermore, a differentiation can be made between “problem-focused” and “emotion-focused” approaches [24,28]. In the former, an attempt is made to control or change the stressor itself. In the “emotion-focused” approaches, on the other hand, the stressor itself is not addressed, but the regulation of the emotional effects of the stressor is aimed at [28]. Individual coping strategies can lead to both adaptive and maladaptive adjustment processes in the context of different stressors [29].

The aim of the present study was to investigate the specific coping strategies and the occurrence of positive changes in the sense of PTG in a sample of TIA patients using a qualitative approach. For this purpose, semistructured interviews were conducted and content analyzed.

## 2. Materials and Methods

### 2.1. Study Design

This qualitative study was originally embedded within a larger, prospective, longitudinal observational cohort study, containing both a quantitative and a qualitative part. The results of the quantitative part have already been published in 2020 [7]. In the context of the longitudinal observational study, consecutive TIA patients admitted to the stroke unit of the Department of Neurology of the University Hospital of Heidelberg were included between May 2016 and June 2017. For the qualitative investigation, semistructured interviews were conducted with TIA patients three months after TIA. For this purpose, patients were consecutively recruited from the cohort study collective at the follow-up time. Recruitment was conducted until content saturation of the qualitative data was guaranteed. In the literature, a number of approximately 12 subjects is assumed to achieve sufficient saturation of qualitative data [30].

### 2.2. Patients

TIA was defined in accordance with the 2009 published AHA/ASA (American Heart Association/American Stroke Association) statement as a transient episode of neurological dysfunction caused by focal brain ischemia without infarction, persisting for 24 h or less [3]. Neurological dysfunction with more than 24 h duration was not classified as TIA. All patients underwent a CT scan or MRI scan; those with ischemic brain injury leading to the current symptoms were not classified as TIA. Inclusion criteria were sufficient German language skills and the ability to remember the TIA event. The presence of PTSD symptoms was not an inclusion criterion. Patients with dementia, delirium, psychotic disorders or severely disabling comorbid somatic disorders that inhibited patients to fill out questionnaires were excluded.

### 2.3. Development of Semistructured Interview Manual

The manual for the semistructured interview was developed following the guidelines for qualitative content analysis published by Mayring [31]. Mayring’s procedure corresponds to a content analysis approach with both deductive and inductive strategies. In the first step, a simple interview manual was designed based on the research question and the scientific background. Using this preliminary manual, interviews were conducted with four patients, tape-recorded, and subsequently transcribed. Two members of the research group (VGH and CN) reviewed the first four interviews. The final interview manual was then created based on the corresponding findings (see Table 1). The final interview manual was used to conduct all subsequent interviews.

### 2.4. Procedure

Interviews were conducted by an experienced interviewer (VGH) three months after the TIA by telephone or face-to-face contact in the outpatient clinic of the Department of General Internal Medicine and Psychosomatics of the University Hospital Heidelberg. The interviewer regularly received feedback and support from experienced colleagues (CN and DK). All interviews were tape-recorded and subsequently transcribed verbatim for qualitative content analysis. This is a pragmatic approach to qualitative analysis that enables to search for overarching categories and themes in a given data set. The qualitative content analysis aimed to generate clusters within the content of the interviews, indicating the possible changes in the aftermath of a patient’s TIA and their way of coping with those changes. Our specific research question is reflected in the interview questions, as well as in the categories and themes which were generated in the qualitative content analysis.

### 2.5. Data Analysis

The mean age, gender distribution, and prevalence of patients with a history of stroke or TIA were calculated using IBM SPSS version 24 statistical software [32]. The analysis was performed according to the guidelines for qualitative content analysis [31]. In the first step, all transcripts were read and emerging themes were coded. Subsequently, content-related themes were grouped into categories. The resulting category system was revised by two experienced researchers (CN and VGH), who discussed and adjusted the grouping of main categories, categories, and themes. The final category system was then used to code all transcripts a second time. All steps of the qualitative content analysis were performed using MAXQDA 2018 software [33].

### 2.6. Ethics

The study protocol was developed according to the STROBE guidelines for observational studies [34] and in accordance with the Helsinki II declaration [35]. Prior to the recruitment of patients, the study was approved by the independent ethics committee of the Medical Faculty of Heidelberg University (S-231/2016). Written informed consent was obtained from all study participants.

## 3. Results

### 3.1. Patient Characteristics

As described above, the present study was originally embedded within a larger, prospective, longitudinal observational cohort study, containing both a quantitative and a qualitative part. The results of the quantitative part have already been published in 2020 [7]. Of *n* = 61 patients, which were investigated at follow-up within the larger longitudinal study, we asked *n* = 20 individuals to participate in our qualitative interview study. Three patients declined to participate, and *n* = 17 patients consented. The average interview duration was 22 min (IQR 11–27). The median age of participants was 66 years (IQR 56–76); 70.6% were male. Approximately one-third of the patients had experienced a TIA before the current TIA; overall 11.8% had experienced a previous ischemic stroke.

### 3.2. Main Categories, Categories, and Themes Derived from Qualitative Analysis

With *n* =17 interviews, the main categories, categories, and themes resulting from the qualitative content analysis gradually reached sufficient content saturation. In the following, the results of the 17 semistructured interviews with TIA patients three months after TIA are presented. The resulting main categories, categories, and themes are summarized and presented with quotations. Through qualitative content analysis, 332 codes were identified, from which a total of 3 main categories, 7 categories, and 36 themes were generated. Table 2 gives a semiquantitative overview of the resulting main categories, categories, and themes.

### 3.3. Main Category A “Impairments as a Consequence of TIA”

A majority of TIA patients mentioned at least one impairment related to the TIA that had occurred during the three-month follow-up interval. Within the main category the (sub-)categories “Physical impairments,” “Negative emotions,” and “Cognitive impairments” were identified (see Table A1, Appendix A).

#### 3.3.1. Category: Physical Impairments

The physical impairments most frequently reported by TIA patients as a result of the TIA were symptoms that patients classified as side effects of new medications, such as increased bleeding tendency, dizziness, and fatigue. In addition, almost half of the TIA patients reported recurrent neurological deficits that occurred one or more times and were symptomatically mostly similar to TIA (difficulties in word-finding, visual disturbances, sensory disturbances, or dizziness). Furthermore, TIA patients complained of general fatigue, dizziness, sleep disturbances, and sexual dysfunction, among other symptoms.

#### 3.3.2. Category: Negative Emotions

Patients often reported negative emotions, which occurred as a result of the TIA. Some patients indicated unpleasant thoughts about a possible recurrence of TIA or of a stroke, which were partly associated with depressive symptoms. In some cases, the depressive feelings subsequently led to the beginning of outpatient psychotherapy. However, the majority of patients’ moods had improved significantly at the time of the interview. Aside from that, financial and occupational worries were indicated by the patients due to ongoing excessive demands in the job or the loss of a job. Another theme was the presence of feelings of failure. The affected patients reported professional achievements before the TIA and felt set back by the severe lack of energy after the TIA. Eventually, some interviewees also reported anger and feelings of powerlessness when realizing that they could no longer take over certain activities at their respective jobs and that other people had to fill in for them.

#### 3.3.3. Category: Cognitive Impairment

Some TIA patients reported cognitive impairments, which occurred as a consequence of the TIA. In part, participants described difficulty concentrating, for example, during conversations or while watching television. In addition, TIA patients reported increased forgetfulness and inattention in everyday life. In two patients, the cognitive impairments had already improved at the time of the interview; one patient reported persistent impairments.

### 3.4. Main Category B “Coping Strategies”

The coping strategies described by TIA patients in dealing with problems and complaints as a result of TIA resulted in the two categories “resource activation” and “avoidance”. These two categories are described below (see Table A2, Appendix A).

#### 3.4.1. Category: Resource Activation

Some of the interviewed patients reported (re-)activating their own resources in dealing with problems and complaints as a result of the TIA. In this regard, most patients sought support from the health care system and frequently reported physical check-ups (carotid ultrasound, cardiac examinations, Otolaryngologist examinations, and nerve conduction velocity measurement), as well as consultations with physicians for reassurance. Two patients reported that they had been prescribed medications to treat health problems that had occurred after the TIA incident. Both patients had received medications for dizziness; one patient had also received psychiatric medication to treat a major depressive episode. The same patient (M, 55 years) had been treated as an inpatient in a neuropsychiatric hospital for a month for depression that had developed after the TIA incident and was undergoing outpatient psychotherapy at the time of the interview. Two patients (M, 68 years, and W, 61 years) indicated that they intended to apply for inpatient rehabilitation treatment from the pension insurance company for problems that had developed after the TIA. One patient had been denied rehab treatment, and the other patient was planning to apply for rehab treatment at the time of the interview. Other resources reported by TIA patients were nature, exercise, work, and family. Fresh air and closeness to nature helped three patients cope with post-TIA problems. Furthermore, two TIA patients stated that exercise helped them to cope with stress or recurrent sensory disturbances. One patient reported that getting back to work quickly helped her regain better concentration and performance. One patient reported receiving frequent support from his family.

#### 3.4.2. Category: Avoidance

A few patients reported avoidance behavior towards thoughts of the TIA or subsequent problems. This involved trying to forget the TIA incident or distracting oneself in challenging situations. Two patients also reported addictive behaviors in dealing with problems that had arisen as a result of the TIA: One patient stated that she liked to smoke a cigarette to relax; another patient stated that she had started gambling after the recent TIA incident and that this was one of the few ways for her to calm down.

### 3.5. Main Category C “Posttraumatic Growth”

A majority of TIA patients reported one or more positive changes as a result of the TIA. The categories “Increased self-care” and “Changed value system” that emerged in the analysis are presented below (see Table A3, Appendix A).

#### 3.5.1. Category: Increased Self-Care

Some TIA patients reported behavioral changes as a result of the TIA that involved an increase in self-care. In this context, TIA patients frequently reported improved perception, implementation, and communication of their own limits. These processes subsequently affected several areas of life. Two patients made long-term career decisions as a result of the TIA with the goal of reducing their workload. Other participants reported “slowing down” overall and taking more breaks. One patient emphasized that the TIA taught her to say “no” and indicated that she was grateful for having learned this. Other changes that affected self-care were lifestyle changes: some patients reported that they had changed their diet after the TIA, eating fewer carbohydrates, less fat, or more fruits and vegetables, or had implemented more exercise into their daily routine since the TIA. Occasionally, TIA patients reported that they drank less alcohol or smoked fewer cigarettes since the TIA.

#### 3.5.2. Category: Changed Value System

Some patients reported changes in their behavior or in the way they perceived life that could be interpreted as a change in their value system. In this context, TIA patients reported that they valued relationships with other people more positively again, had developed an increased awareness of spirituality, or generally tried to value positive things in their own lives more. This section may be divided into subheadings. It should provide a concise and precise description of the experimental results, their interpretation, as well as the experimental conclusions that can be drawn.

## 4. Discussion

In the present study, the subjective experiences of TIA patients were investigated. Seventeen TIA patients were asked three months after the TIA which problems they had experienced as a result of the TIA, how they had dealt with them, and whether they had noticed positive changes as a result of the TIA. In the following, we have structured the discussion of our results in accordance with the three main categories, identified by the thematic content analysis of the interviews.

### 4.1. Impairments

Overall, 15 of the 17 TIA patients reported at least one impairment that had occurred as a consequence of the TIA. Most frequently, TIA patients described physical impairments, followed by negative emotions and cognitive impairments. In the context of physical impairments, seven patients indicated recurrent TIA symptoms in the three-month period after the primary incident, such as sensory, visual, or speech disturbances. Against the background that TIA is a risk factor for both a major stroke [5,6], and the occurrence of recurrent TIA symptoms, many patients in our study reported fear of recurrent TIA or even stroke. Previous studies have shown that fear of stroke following TIA is a common phenomenon in TIA patients [22,23,36,37]. In addition, two patients in our study also reported depressive symptoms. Different hypotheses have been proposed to explain the association between a stroke and the subsequent development of depression. On the one hand, psychobiological mechanisms have been discussed as factors for depression after a stroke [38]; on the other hand, depression could also be facilitated as a consequence of functional or cognitive limitations in daily life [37]. The occurrence of depressive symptomatology after TIA has also been reported by previous qualitative studies [23,37]. Concerning recurrent TIA symptoms less attention has been paid to this in clinical practice and especially in communication with the patient. In this context, a more focused education of TIA patients about a possible recurrence of TIA symptoms and recommendations for subsequent action may relieve patients and contribute to a better understanding and management of the disease.

In total, ten TIA patients reported medication side effects in the interviews. The side effects are often related to antiplatelet or antihypertensive medication, which were prescribed for secondary prevention. They were subjectively experienced as impairing in different ways, for example in the form of an increased bleeding tendency, dizziness, or fatigue, which in some cases subsequently led to further consultations with physicians. In previous studies, mainly the bleeding rate under treatment with antiplatelet drugs, such as aspirin, was investigated in stroke patients [39,40]. However, no studies on the subjective experience of medication side effects after stroke or TIA have been published yet. In particular, a negative correlation between experienced medication side effects and medication adherence can be assumed. Tedla and Bautista (2016) investigated the association between the side effects of antihypertensive medication and adherence and were able to demonstrate that side effects led to lower medication adherence [41]. In particular, excessive urination and decrease in sexual drive seem to significantly predict lower adherence [41]. In a study by De Schryver et al. (2005), determinants of non-adherence to aspirin or oral anticoagulants in stroke patients were investigated. In this study, older age and higher aspirin dosage were associated with drug non-adherence, while the occurrence of dizziness was associated with higher adherence to aspirin [42]. In view of the importance of effective secondary prevention after TIA, future studies should specifically focus on the effects of physical impairments, fear, and depression on medication adherence.

### 4.2. Coping Strategies

In the qualitative analysis of the interviews, two different coping styles could be identified: “resource activation”, as a type of active, partly problem-focused coping style; and “avoidance”, as a type of passive coping style. Overall, nine of the seventeen participants reported resource activation in dealing with discomfort and impairment after TIA. The most common category mentioned by TIA patients was actively seeking support from the health care system. In this regard, eight TIA patients sought medical support or consultations because of problems that arose as a result of the TIA. In some cases, this involved re-examinations for risk evaluation and prevention of further cardiovascular events. The patients reported that additional examinations and especially the conversation with the physician had had a calming and reassuring effect. Furthermore, TIA patients felt supported by medications, psychotherapy, and other people with health care professions. In the interviews, eight TIA patients reported benefiting from support services offered by the health care system. In addition to offers of help from the health care system, TIA patients also drew on their own resources. In this respect, nature experiences, and bodily exercise were reported frequently as helpful measures. One patient received support from family and another patient actively sought after employment and challenges at work. Previous studies already investigated the effects of psychosocial interventions to improve coping with stress after TIA or a stroke. In a systematic review, Lawrence et al. (2013) demonstrated a high benefit of mindfulness-based interventions for TIA/stroke patients in terms of psychosocial outcomes such as anxiety, depression, mental fatigue, and quality of life [43]. However, studies on the impact of personal resource activation on the stress experience of TIA patients have not been published yet.

In total, five of the 17 TIA patients reported behaviours of avoidance in dealing with the TIA or subsequent problems. By avoiding thoughts, feelings, and memories, these patients indicated to have partly achieved a higher sense of well-being. TIA patients often experienced their own avoidance behaviour as positive, equally as a successful strategy to deal with the stressful situation. Two patients also reported addictive behaviour in dealing with discomfort and impairment after TIA: One patient stated that he prefers to smoke a cigarette to calm down after experiencing post-TIA impairments. Another patient reported that she had started gambling as a result of the TIA and accompanying problems. Avoidance in terms of coping is often described as maladaptive because it does not lead to long-term change. Maladaptive coping strategies can furthermore have a negative impact on medication adherence. In a systematic review by Zwikker et al. (2014), an association between an avoidance coping style and medication non-adherence was demonstrated [44]. Vice versa, an active coping style seems to be a predictor for medication adherence [45,46]. On the other hand, the presence of maladaptive coping styles has already been shown to be a predictor for PTSD severity three months after the TIA [47]. In a recent study, Assayag et al. (2022) similarly examined risk factors for the development of PTSD after stroke and found that high-anxious and defensive coping styles were associated with a six-fold higher risk for poststroke PTSD in comparison with low-anxious and repressive coping styles [48]. However, in the context of our finding that TIA patients reported an avoidance coping style, it must be considered that the phenomenon of avoiding feelings or thoughts can also be regarded as a cardinal symptom of PTSD, which indeed was demonstrated in 24.6% of the cases in the population we studied [7].

In summary of the discussed studies, it becomes clear that no uniform classification or definitions of coping styles are used, so the results can only be compared with each other to a limited extent. However, it becomes clear that maladaptive coping strategies have a negative influence on the course of the post-TIA phase and possible symptoms of PTSD in several respects.

### 4.3. Posttraumatic Growth

In the present study, 14 of the 17 TIA patients reported at least one positive change that had occurred as a result of the TIA. In our thematic analysis, two categories emerged: “Increased self-care” and “Changed value system”. TIA patients particularly stated that they had become more aware of their own limits since the TIA, and subsequently tried to increasingly withdraw from stressful situations; this concerned situations at work, but also social situations. Furthermore, eight patients indicated that they attempt to implement rest breaks more often in their everyday life since TIA. Previous literature on posttraumatic growth rarely described becoming more aware of one’s own limits and the more frequent implementation of rest breaks as a consequence of having experienced TIA. It is possible that this form of posttraumatic growth occurs particularly as a result of physical illness since physical illness is often considered to be a long-term consequence of working overload and stress in everyday life. However, studies confirming this interrelation are currently not available. Furthermore, five TIA patients reported a change in diet, an increase in physical activity, a reduction in nicotine and alcohol consumption, and improved medical compliance. These aspects of increased self-care after TIA have already been identified by previous qualitative studies [22,23]. In these investigations, TIA patients reported increased health awareness and lifestyle changes after TIA, such as a healthier diet and more physical activity [22,23]

In total, four TIA patients reported changes in their own value systems. In this regard, TIA patients described valuing interpersonal relationships, spirituality, and nature experiences more after the TIA. These described changes can be seen as typical examples of posttraumatic growth [14]. The present study is the first to demonstrate such positive changes in the value system as a possible consequence of experiencing a TIA. To the best of our knowledge, no quantitative studies focussing on the occurrence of particular PTG after TIA have been published to date. However, there are a few studies that have investigated the phenomenon of PTG after experiencing a *stroke*. In a study from 2021, Sherratt and Worrall examined a group of patients who suffered from aphasia as a result of a stroke. They found that some of the patients had developed PTG in the first year after the stroke and had managed to redefine themselves in a positive way, in the context of their limitations [49]. In a qualitative study by Kuenemund et al. (2016), an increased appreciation of life and more intense relationships could be identified as the most common positive changes after TIA [21]. Previous studies have also shown that PTG after stroke is inversely correlated with depression and anxiety [19,50]. This association was indicated to become statistically stronger the more time had passed since the stroke [19]. Social support and deliberate rumination were shown to be important predictors of the development of PTG after stroke [51].

As described above, previous studies indicated that PTG in the context of neurological disorders may increase over time [16,17]. Since one-third of the patients in our study had suffered a previous TIA and 11.8% had suffered a previous ischemic stroke before the current TIA, it can be assumed that in these patients PTG might have developed already before the current TIA. However, this assumption would have to be investigated in further studies. In addition, it remains unclear which factors are more likely to lead to PTSD or more likely to lead to PTG in patients after a TIA/stroke. In their 2016 study, Kuenemund et al., therefore, hypothesized that both negative and positive identity changes, in terms of PTG, may occur after a stroke or TIA [21]. In summary of the results of our thematic analysis, PTG after TIA appears to be a common phenomenon, which should therefore be considered in the treatment and rehabilitation of TIA patients.

### 4.4. Limitations

Several limitations of the presented study should be addressed: All TIA patients who participated in the interviews had also taken part in the quantitative cohort study which may have induced increased reflection on their own experience shortly after the TIA. Possibly, the TIA patients, therefore, dealt more consciously with changes and problems that arose after the TIA. In addition, it should be considered that a selection bias may have arisen due to the initial exclusion and the dropout rate. Furthermore, the interviews represent a qualitative approach, which may be generally susceptible to biases, such as the influence of the interviewer. We tried to counteract these possible effects by using an experienced interviewer who received feedback and support from experienced colleagues. However, we cannot rule out that some relevant aspects in the context of coping strategies and posttraumatic growth in TIA patients may not have been mentioned in the interviews.

## 5. Conclusions

Three months after TIA, patients can suffer from various physical impairments, which involve medication side effects and recurrent neurological symptoms, partly causing fear about the recurrence of a TIA or the occurrence of a stroke. These physical impairments may result in reduced adherence to medication or lead to the development of post-TIA depression. As a common coping strategy, TIA patients reported activating available resources, such as the health care system or enjoying nature, performing physical exercises, or spending more time with family members. However, a proportion of patients also indicated avoiding negative thoughts and feelings, which can be classified as a more passive, and possibly maladaptive coping strategy. Establishing maladaptive coping mechanisms after TIA could subsequently lead to further health problems or even promote the recurrence of cerebrovascular events, due to persistently high levels of stress or reduced adherence to medication. Therefore, we suggest that TIA patients should receive regular basal psychosocial support to develop individual, adaptive coping strategies with the help of psychosocially trained personnel.

In our thematic content analysis, a majority of TIA patients reported at least one positive change that had occurred as a result of the TIA. In this context, patients reported increased self-care and a positively changed value system. Thus, posttraumatic growth seems to be a common phenomenon after TIA, which may have important implications for treatment and rehabilitation. In summary, the present study points to the importance of basal psychosocial care for patients after TIA with the aim of developing adaptive coping strategies and promoting PTG, which could play an important role in preventing recurrent cerebrovascular events.

## Figures and Tables

**Table 1:** Interview manual for the semistructured interview with TIA patients three months after TIA.

Main Open Question		Encouraging Question
1.	How have you been since the TIA? Have you noticed any changes compared to the time before the TIA?	Have there been changes concerning… - Body? - Medication + side effects? - General Condition/mental health? - Profession/Household/Hobbies? - Relationships? - Spirituality?
2.	How did you deal with the changes?	- What helped with problems? - What has changed in your behavior? - What has changed in your attitudes?
3.	Were there any positive changes (other than those already mentioned), and if so, what were they?	

**Table 2:** Main categories, categories, and themes resulting from semiquantitative analysis of interview statements from TIA patients three months after TIA (*n* = 17). In round brackets, the first number: Number of TIA patients, who named aspects of main categories, categories, and themes. In round brackets, the second number: Number of the corresponding codes.

Main Category A “Impairments as a Consequence of TIA” (15; 99)	
**Physical impairments (15; 75)** Drug side effects (10; 17) Bleeding tendency (4; 7) Drowsiness/dizziness (2; 3) Headache (1; 1) Fatigue (1; 3) Skin rash (1; 2) Shortness of breath (1; 1) Recurrent neurological deficits (7; 20) Speech disturbances (3; 7) Visual disturbances (3; 5) Sensory disturbances (3; 5) Dizziness (3; 3) Fatigue (4; 18) Dizziness (3; 7) Sleep disorders (2; 2) Sexual dysfunction (1; 2) Weight gain (1; 4) Headache (1; 1 Tinnitus (1; 2) Pain (1; 2)	**Negative emotions (8; 14)** Fears/worries about the recurrence of TIA or stroke (5; 5) Depressive symptoms (2; 3) Financial worries (2; 2) Anger (1; 1) Feelings of failure (1; 2) Powerlessness (1; 1) **Cognitive impairment (3; 10)** Difficulty concentrating (3; 6) Forgetfulness (2; 3) Inattention (1; 1)
**Main Category B “Coping Strategies” (10; 50)**	
**Resource activation (9; 43)** Healthcare system (8; 28) Check-ups (4; 6) Discussions with physicians (4; 7) Medications (2; 5) Application for rehab (2; 2) Psychotherapy (1; 5) Chiropractor/health practitioner (1; 3) Nature (3; 6) Exercise (2; 3) Work (1; 2) Family (1; 4)	**Avoidance (5; 7)** Distraction (2; 2) Suppression (2; 2) Addictive behavior (2; 3)
**Main Category C “Posttraumatic Growth” (14; 70)**	
**Increased self-care (12; 55)** Maintaining one’s own limits (8; 32) Change in diet (5; 11) Increased physical activity (3; 7) Reduction of nicotine consumption (1; 3) Reduction of alcohol consumption (1; 1) Compliance with support stockings improved (1; 1)	**Changed value system (4; 15)** Appreciation of relationships (3; 4) Increased spirituality (2; 6) Increased awareness of positive things in life (1; 5)

## Data Availability

The datasets used and analyzed during the present study are available from the corresponding author upon reasonable request.

## Appendix A

**Table A1:** Example quotes on the themes and categories within the main category A “Impairments as a Consequence of TIA”.

Main Category A “Impairments as a Consequence of TIA”
**Category “Physical Impairments”**
**Medication side effects**
“The first time was of course with the ASS 100. When I hit myself somewhere, of course then I bleed like crazy. That is also just such a side effect.” (W, 61 years)
“[…] that I cut my hand with scissors in such a way that the blood just ran out. But then I squeezed everything shut and everything was fine again. But the blood is noticeably thinner since the aspirin.” (M, 90 years)
“Dizziness, I think, at least, that it [comes] from the medication–because before I never had something like that with the other medications.” (M, 84 years)
“Because of the medication [I] also [have] the tiredness, that‘s clear, but they also told me, that’s because of the beta blocker. And, so [I suffer] the fatigue above all. Until the body gets used to it, I would say.” (M, 34 years)
**Recurrent neurological deficits**
“Yes, I have the words in my head, but I can’t say them. Because I can’t find them. Or I can’t get them out. (…) I know exactly what I want to say, but I can’t. (..) It has [been] since sometime last week.” (W, 61 years)
“Yes, for example, if I had had the conversation we’re having now, let’s say two months ago, we wouldn’t get very far because I have ongoing word-finding problems–would have–would have had” (W, 56 years)
“Yes, actually twice more. So short–yes, I’ll say speech problems. Once, when I was out with my acquaintance, we went for a walk, and I wanted to say something, and–didn’t go right again. But only very briefly. And then the same situation happened again. Yes–what should I do there? Then–yes, then I just think, yes good, it was only very briefly, and now it’s gone again–and well.” (W, 75 years)
“It was only once like this–I did not feel dizzy, but, how can you say, I sat at the computer and suddenly both sides were black.” (W, 62 years)
“So I was practically sitting at the table, and saw my friends who were sitting on my left, I suddenly couldn’t see them anymore. But I could just see straight ahead.” (M, 69 years)
“Yes, at the beginning I had such a strange electric shock with my left hand, when I stretched it out, the index finger and the middle finger always had such a strange electric shock when I stretched it out.” (M, 34 years)
“Yes, so like an ant, an ant tingling in my arm and foot.” (M, 68 years)
“Also because of the dizziness…because I didn’t have that before, I always thought, what is this dizziness now.” (M, 34 years)
**Fatigue**
“So, what I’ve had after being discharged was this fatigue, so it took me a very long time. I could be up during the day–I could be up for two hours, then I had to lie down again, it took a very long time.” (W, 44 years)
“I also get tired faster, exhausted faster. I notice that at work right now, too.” (M, 34 years)
“So that was so, which is now also difficult, physical resilience was zero, you really have to say zero, I more or less crawled from chair to chair, so there was no energy left at all.” (W, 56 years)
**Dizziness**
“At the beginning of the time, I often felt dizzy, so I couldn’t do much now.” (M, 68 years)
“I then had another slight dizziness afterwards […] Yes, that was, let’s say a spinning dizziness.” (M, 78 years)
**Sexual dysfunction**
“[Concerning] sexual contact nothing has worked, I had, as I said, a long-distance relationship […]. And, yes, when she came, I didn’t think about it, I thought it would be like before. When she came, I just saw that nothing works anymore. And, yes, she was also shocked, me too, of course, because I didn’t think it wouldn’t work.” (M, 34 years)
**Category “Negative emotions”**
**Fears/worries about a recurrent TIA or stroke**
“[…] Because they just said it was a circulatory disorder, and, actually, nothing was found at all, and sometimes you think, was it something, or, will it come again, or so?” (M, 69 years)
“The only thing where I’m a little afraid is when you–um, for example, when jogging, and if one does that somehow alone, then I’m a little worried that one might fall over there now.” (W, 72 years)
“Every now and then I just thought about it, hopefully, it won’t happen again.” (M, 68 years)
**Depressive symptoms**
“So […] there was […] a medium depression […]on top of it, on top of the whole thing. […] and then mood swings. From […] just totally down in the dumps to […] what one would call a normal mood.” (M, 56 years)
“And that has me of course also depressed, logically, because usually, I am a smart woman, and that was then already depressing, uh, […]” (W, 44 years)
**Financial/professional worries**
“[…] that the same thoughts don’t come up again and again, again and again with me: how’s it going with the job, how’s it going with the job, how’s it going […] professionally.” (M, 56 years)
“Yes, so, Christian, my husband, then also said, when I [was discharged] from the hospital, where he […] picked me up, and [he] said: we give up the store. And then I suddenly panicked even more, then I said, what do you want to give up the store, should I now knit socks or what. That’s not possible at all. That doesn’t work at all.” (W, 61 years)
**Feelings of failure**
“And because I was just, let’s call it the flippant workhorse before, and then I was slowed down to zero, I had initially seen that as, uh, can we say, personal failure.” (M, age 56)
“[I] can’t do it anymore. Yes exactly, that’s the problem. […] And that annoys me, that gets me down, that makes me sick. I try to do that again and again, and I have great staff who take everything out of my hands again and again, but (…) that’s even worse for me. Because then I’m shown again that you can’t do that.” (W, 61 years)
**Category “Cognitive Impairments”**
**Concentration difficulties**
“Yes, concentration was very difficult, um, sometimes just grasping the correlations, well, that was already very delayed. I already noticed that. […] I was always told, when I talked, that I spoke very slowly, with a lot of deliberation, but that was because I couldn’t grasp things that were said to me so quickly. So, I already had a delay in that, too.” (W, 44 years)
“Concentration and retentiveness were strongly impaired. […] and I couldn’t watch TV because my concentration wasn’t there.” (M, 56 years)
“And what is also very bad for me is that since I was with you in the head clinic, I was able to listen to three people, four people before, when now, when I do something, or […] someone tells me something, and then someone else joins in and someone else joins in, then my ears […] are ringing. Simply overwhelmed, I feel overwhelmed.” (W, 61 years)
**Forgetfulness**
“So that has, that was also a shortcoming. That I noticed […] that I […] that I, that I forget a lot.” (W, 61 years)
“And, just so with the retentiveness and concentration, was also very low.” (M, 56 years)
**Inattention**
“So […], since I’m out of the clinic, [I have] become a scatterbrain, I run everywhere again, I bang against somewhere again, I always tear something. Even my husband has said that I’ve become really scatterbrained.” (W, 61 years)

**Table A2:** Example quotes on the themes and categories within main category B “Coping Strategies”.

Main Category B “Coping Strategies”
**Category “Resource Activation”**
**Health system**
“An ultrasound was done of the carotid artery because it has stenosis on the right side, and so an ultrasound was done again, that showed that this has not really become more severe. […] they also said that everything is still perfectly fine.” (W, 61 years)
“[…] then I was at the doctor, then at the whole heart examination and clinic, then I was also […] at the neurologist […] who then tested my arm, everything was actually ok then. They do such electrical tests then.” (M, 34 years)
“I went to Dr. M… on Tuesday, and he confirmed that nothing has worsened [in the carotid stenosis].” (M, 68 years)
“[The dizziness] is explained, so [the] Otolaryngologist [said], there was nothing, and that’s why they then also attributed it as being psychosomatic.” (M, 55 years)
“The [doctor] also went through all the reports again […], and he also said, yes well, that’s all you can do.” (W, 61 years)
“Because when he gave me the certainty, as I said, that I will not get 99.9% through the medication, it was actually clear to me that this was only psychological then.” (M, 34 years)
“I have medications […] on the list, plus a new one, it’s called Abilify, 5 mg, so the smallest dosage […].” (M, 55 years)
“That’s where I get medication now, too, for the dizziness.” (M, 55 years)
“That was, let’s say, a kind of spinning dizziness. It went away, I went to a cardiologist here, and he prescribed me some more drugs.” (M, 78 years)
“[…] and then I would like to apply for […]rehab. […] Just for myself, just for myself.” (W, 61 years)
“Yes, yes. So, what annoys me a bit is, and that is–I wanted to do a rehab, yes. […] And then they rejected it from the health insurance.” (M, 68 years)
“I was […] an inpatient in the neuropsychiatry for one month, then 6, 8 weeks day clinic, and now I am in the outpatient department. […] That means I have a conversation with the psychologist every 3 weeks. […] Now that it is clear to me that depression is an illness, I can deal with it much better. […] To understand that this is not a failure, this is an illness, triggered by various factors.” (M, 55 years)
“Yes, […] he did […] a lymphatic massage that I had once and he just pressed [some] points, here on the neck somewhere, there where it goes up there, and that also helped me, I must say.” (M, 78 years)
**Nature**
“The climate above all, the air. […] And then in the evening I walked along the sea there, just fresh wind.” (M, 34 years)
“[Then] I went out into the air. […] When I got out into the air, when I got out, it got better [the dizziness].” (M, 78 years)
“So what has built me up is actually that the weather has become better recently and […] when I get bad, I say, come on, let’s go out into the garden for an hour. That helps me a lot, because we still have, um, I’m quite proud, […] a huge garden with a pool, and there is no one, and there I come to rest.” (W, 61 years)
**Exercise**
“When I realized this [the sensitivity disturbance], I got up right away and walked around the apartment.” (M, 34 years)
“I applied for this rehabilitation sport. I am trying to do rehabilitation sports and with this maybe […] I’ll get a kick, to say, with that, I can get rid of my fear.” (W, 61 years)
**Work**
“So, the best medicine I prescribed for myself was simply to say again, I’m going to start working again, I need that for my head, I need work so that I can do something because being at home for a long time is not for me.” (W, 44 years)
**Family**
“My family helped me a lot. Especially [my family] in Turkey. […] I could, I say, really switch off, really. Not worry about anything.” (M, 34 years)
**Category “Avoidance”**
**Repression**
“I mean, you just repress it. So bad things like that I try to forget really quickly. Such negative things.” (M, 77 years)
“I just don’t want to know about it, you know, like that. Let’s say, and by not dealing with it, it somehow disappears again.” (M, age 84)
**Distraction**
“I’ve had tinnitus on the left side […] since I’ve had the circulatory disorder on my head. Which means, I had it 5, 6 years ago, then it went away, and now it is again intensified, partially, […] well, I don’t always think about it, [instead I use] distraction.” (M, 68 years)
“So when I feel bad, I say, ’come on, let’s go into the garden for an hour. […] And the mental aspect, […] so great, feels good, I think of nothing and can just calm down.” (W, 61 years)
**Addictive behavior**
“Yes, I can also switch off, drink coffee, have a smoke.” (M, 34 years)
[Patient tells that she started gambling] “I told him [the family doctor] once a few weeks ago that I gamble.” (W, 61 years)

**Table A3:** Example quotes on the themes and categories within main category C “Posttraumatic Growth”.

Main Category C “Posttraumatic Growth”
**Category “Increased Self-Care”**
**Maintaining one’s own limits**
“[…] but no matter what kind of job comes along, what I’m striving for, at least, is that I don’t want to let myself get pushed into this vicious cycle anymore.” (M, 54 years)
“[…] that I have changed, let’s say, maybe in my whole attitude, in my way of life a little bit. Not doing as much as before, allowing myself more rest.” (M, age 81)
“Now when I notice I‘m, well I‘m, I‘m getting tired, well not tired, but knackered, then I just take the break.” (m, 34 years)
“[…] that now I also allow myself to rest and also say sometimes, “No, I don’t do it”, “No, I don’t feel like it”, that’s something great for me, something absolutely great because it really took me 43 years to do that now–I really have to say–I’ve definitely taken that with me, in any case.” (W, 43 years)
**Change of diet**
“My wife and I don’t eat seafood anymore. (Laughs) Yes, we were in Elba, no mackerel, no mussels–so this cholesterol thing. We pay much more attention to that now. […] definitely–so since June I haven’t eaten any mussels, no crabs, nothing at all.” (M, 59 years)
“Yes, I now also eat an apple in between meals, I wouldn’t have done that before.” (w, 44 years)
“I have now actually resolved that I […], must now pay more attention to it, I have before probably lived quite normally, I have not had a dissolute lifestyle, […] but now I actually give more attention to it.” (M, 90 years)
“I’m eating–well from a dietary point of view I have to say, I’ve been eating healthier for the last four months. I definitely have to say that.” (M, 59 years)
**Increased athletic activity**
“The only thing that has changed is that I get on the exercise bike several times a week.” (M, 69 years)
“So that’s one thing, and the second thing is that I’m more intensively involved with the topic of sport again. […]I’m in a cardiac sports group, the other thing was forbidden to me by the cardiologist, only under medical supervision, but now I go there regularly every week for an hour.” (M, 77 years)
**Reduction of nicotine consumption**
“I used to have a pack, a pack and a half […] But now, I don’t smoke that much, 10 cigarettes maybe a day.” (M, 34 years)
**Reduction of alcohol consumption**
“Yeah, I definitely drink less alcohol, like it was a few months ago.” (M, 73 years)
**Category “Changed Value System”**
**Appreciating relationships**
“The circle of friends had become smaller and smaller [than before the TIA] due to the fact that I was always working. I have now met an interesting person, namely the cab driver who drove me to the clinic every day, so a friendship has really developed.” (M, 55 years)
“For example, I now plan to go back to my old home country […] in May, I still have two brothers there with their families, and yes, that I’ll do it this year […] you shouldn’t put anything off any longer.” (W, 75 years)
“I see how nice it is to walk through the forest with my dog at 6 a.m. and so on, or to go for a walk with my wife.” (M, 55 years)
**Increased spirituality**
“But when you see creation […] it’s something beautiful. When I, as I said earlier, walk through the forest at 6 or 7 in the morning, when the sun breaks through if one also sees that as spiritual […] then there has been a positive development.” (M, 55 years)
“I believe in Allah, yes. […] God. […] I used to have a necklace […] It was meant to keep the bad away from you. […] And I didn’t have it for years. My aunt noticed while we were in Turkey and said ’hey, you have to wear that’[…] so, I have it now.” (M, 34 years)
**Increased awareness of the positive things in life**
“In the beginning, when I came out of the clinic, I wrote notes and put them in a big glass. Successes. So every day, whether it was a small or a big success, whether it was a nice day with my grandchild […] I started doing that for myself […] that I see the positive things again.” (W, 61 years)
